# Vector Encoding of Phylogenetic Trees by Ordered Leaf Attachment

**DOI:** 10.1007/s11538-026-01611-9

**Published:** 2026-03-18

**Authors:** David Harry Richman, Cheng Zhang, Frederick A. Matsen

**Affiliations:** 1 Computational Biology Program, Fred Hutchinson Cancer Center, Seattle, 98109 Washington USA; 2 Mathematics Division, National Center for Theoretical Sciences, Taipei, 10617 Taiwan; 3 School of Mathematical Sciences and Center for Statistical Science, Peking University, Haidian District, 100871 Beijing China; 4 Department of Statistics and Department of Genome Sciences, University of Washington, Seattle, 98195 Washington USA; 5 Howard Hughes Medical Institute, Fred Hutchinson Cancer Center, Seattle, 98109 Washington USA

**Keywords:** Phylogenetics, Binary tree, Tree encoding, 92B10, 05C05, 05C85, 68W40

## Abstract

As part of work to connect phylogenetics with machine learning, there has been considerable recent interest in vector encodings of phylogenetic trees. We present a simple new “ordered leaf attachment” (OLA) method for uniquely encoding a binary, rooted phylogenetic tree topology as an integer vector. OLA encoding and decoding take linear time in the number of leaf nodes, and the set of vectors corresponding to trees is a simply-described subset of integer sequences. The OLA encoding is unique compared to other existing encodings in having these properties. The integer vector encoding induces a distance on the set of trees, and we investigate this distance in relation to the NNI and SPR distances.

## Introduction

Phylogenetics is the study of evolutionary histories, and how to infer such histories from observed data. An evolutionary history is commonly formalized as the data of a leaf-labeled binary rooted tree (a “tree topology”). Inference and analysis of phylogenetic trees is typically done using a tree as a discrete structure.

However, recent work in phylogenetics has begun to consider the use of vector representations of trees. This is motivated by recent advances in machine learning, where vector representations are the standard output of many classes of models. It is also motivated by the desire to represent collections of trees.

The first of the more recent contributions to this literature includes work by Penn et al. (2024) introducing the Phylo2Vec encoding, which is an integer vector representation of a tree topology. This system encodes a phylogenetic tree with *n* leaves as a list of $n - 1$ integers $(a_1, \ldots , a_{n-1})$, where the *i*-th entry satisfies $0 \le a_i \le 2 i - 2$. However, this encoding requires quadratic time to compute. The HOP metric paper (Chauve et al. 2025) introduced a linear-time encoding, but the resulting set of vectors in this encoding is non-convex and rather complicated. Both of these papers investigated the connection between encoding distances and traditional tree metrics.

In this paper, we introduce a simple new method for encoding a tree topology as a list of integers that can be computed in linear time. We call this the “ordered leaf attachment” (OLA) code. The OLA code is a bijection between the set of rooted tree topologies on *n* leaves and a convex set of length $n-1$ vectors defined by a simple set of coordinate-wise inequalities.

The OLA encoding induces a distance on rooted tip-ordered trees. We show that this OLA distance is bounded “on average” by a constant factor of the NNI distance, in a sense that we make precise, see Theorem 4. We also prove an upper bound for OLA distance between trees differing by a single SPR move in terms of tree height, see Theorem 6. A construction based on the OLA code has already proven itself useful in work defining a variational autoencoder on phylogenetic trees (Xie et al. 2025). A distance based on the OLA code has been shown to quickly identify reticulation events in collections of phylogenetic trees (Markin and Anderson 2025).

## Results

### Informal Description of OLA Encoding

Here we give an informal description of the OLA code, which will be sufficient to give intuition and present results. A detailed description and further discussion is given in Sect. 4.

The basic idea behind the OLA encoding is that a single tree, with a total ordering on its leaves, can be thought of as a sequence of trees obtained by adding the leaves one-by-one. In the OLA encoding, the *i*-th entry of the vector records the location where the *i*-th leaf was added to the tree.

**Fig. 1:**
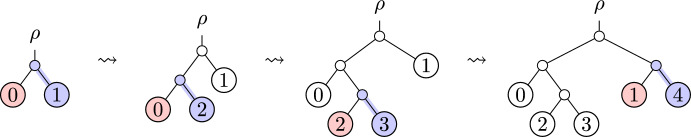
OLA-encoding a Yule-type tree: (0,0,2,1). Yule-type trees are those where each new leaf is added as a sister to a previously-added leaf, and for these trees the OLA encoding simply records the label of the sister leaf. At each step, the new leaf is highlighted in blue, and the sister leaf is highlighted in red

We first consider a special case: say a phylogenetic tree is *Yule-type* if, in the process of building up the tree, each new leaf is added as a sister to a previously-added leaf. (This terminology comes from the Yule random tree model [ Semple and Steel (2003), Chapter 2.5].) For a Yule-type tree, the *i*-th entry of the OLA encoding is just the label of the sister leaf at the step when the *i*-th leaf is added. For example, the tree shown in Figure 1 has OLA code (0,0,2,1).

In the general case, when a new leaf is added, it may be added as the sister of an internal node. Here a complication arises, since the internal nodes do not come equipped with labels. However, using this idea to “record each sister-node label,” the problem of encoding a phylogenetic tree can be reduced to the problem of defining a consistent method for labeling internal nodes of a phylogenetic tree.

**Fig. 2:**
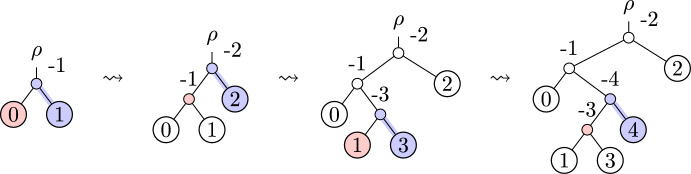
OLA-encoding a non-Yule-type tree: (0,-1,1,-3). For non-Yule-type trees, we introduce a canonical labeling of internal nodes, where each internal node is labeled by $-i$ when the *i*-th leaf is added. Using this convention, we construct the OLA encoding by adding the label of the sister node at each step, whether or not that sister is a leaf. At each step, the new leaf is highlighted blue, and the sister node is highlighted red

Our proposed OLA encoding provides one such labeling of internal nodes. For a tree with *n* leaves, the internal nodes are labeled with negative integers $-1, -2, \ldots , -n + 1$. Each time a new leaf is added to a tree, a new internal node is created. When we add a leaf labeled by *i*, we then label the new internal node $-i$. This gives a well-defined labeling of internal nodes, which we consider “canonical.” An example of these internal node labels is shown in Figure 2. For a larger example, see Appendix B.

### Statement of Bijection

Now we can state the main property of the ordered leaf attachment (OLA) code for a leaf-ordered binary, rooted tree. Let $\widehat{\mathcal {RBT}}_n$ denote the set of rooted, binary trees with *n* leaves equipped with a linear order on the leaf set. Let $\mathcal {C}_{n - 1}$ denote the set of integer vectors

1 $$\begin{aligned} \mathcal {C}_{n - 1} = \{ (a_1, a_2, \ldots , a_{n - 1}) \in \mathbb {Z}^{n - 1} : -i< a_i < i \}. \end{aligned}$$

Note that $\mathcal {C}_{n - 1}$ is *convex*, in the sense that it is the set of integer points within a convex region of Euclidean space $\mathbb {R}^{n - 1}$. We sometimes refer to vectors in $\mathcal {C}_{n - 1}$ as *valid* integer vectors.

We construct an efficiently-computed bijection between $\widehat{\mathcal {RBT}}_n$ and $\mathcal {C}_{n - 1}$.

#### Theorem 1

For any $n \ge 2$, the OLA encoding and decoding algorithms (Algorithms 2 and 3) define a pair of inverse bijections

2 $$\begin{aligned} \Phi : \widehat{\mathcal {RBT}}_n \rightarrow \mathcal {C}_{n - 1} \qquad \text {and}\qquad \Psi : \mathcal {C}_{n - 1} \rightarrow \widehat{\mathcal {RBT}}_n. \end{aligned}$$

#### Theorem 2

The OLA encoding and decoding algorithms have linear time complexity, in terms of the number of leaves.

The proofs of Theorems 1 and 2 are given in the Methods section (Sect. 4), along with the encoding and decoding algorithms. To illustrate one case of this theorem, the OLA encodings of all trees with $n = 4$ leaves is shown in Appendix F.

### OLA Distance and Standard Phylogenetic Distances

The OLA encoding is a bijection between the set of rooted, binary trees with *n* leaves and the set of integer vectors $\mathcal {C}_{n - 1}$. This itself is useful. However, one may wish to understand to what extent similar OLA vectors correspond to similar trees. We introduce a tree-distance based on OLA vectors, and show that it is related to the NNI and SPR distances.

#### Definition 3

The *OLA distance* between two leaf-ordered binary rooted trees $T, T'$ on *n* leaves is the Hamming distance between their OLA encodings.

#### Comparison to NNI distance

We would ideally prefer if the OLA distance were close to a standard phylogenetic distance, for example within a constant factor. Unfortunately, this is not the case when compared with NNI distance.

**Fig. 3:**
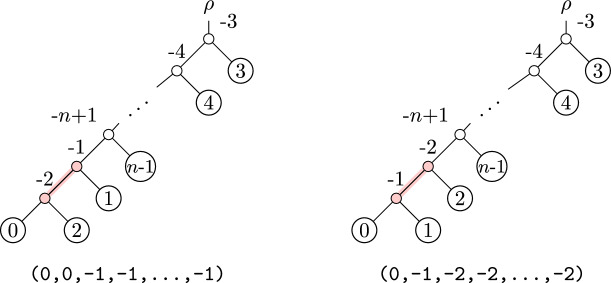
Two trees which differ by a single NNI move, but have large OLA distance

In fact, in the worst case a single NNI move can produce a tree with arbitrarily large OLA distance from some starting tree. See Figure 3 for such an example. This example shows two trees on *n* leaves which are related by an NNI move, modifying the edge highlighted in red. Their OLA codes differ in $n - 2$ entries. This is the largest possible OLA distance between two trees on *n* leaves (since the first entry of the OLA code is always 0).

However, as we show next, the expected OLA distance between two trees which differ by an NNI move is constant in expectation, when averaged over all moves from a given starting tree.

##### Theorem 4

Suppose *T* is a fixed phylogenetic tree in $\widehat{\mathcal {RBT}}_n$, and let $T'$ be a random tree which differs from *T* by an NNI move, chosen uniformly. Then the expectation of the OLA distance between *T* and $T'$ has the constant upper bound

$$ \mathbb {E}(d_{{{\,\textrm{OLA}\,}}}(T, T')) \le 4. $$

**Fig. 4:**
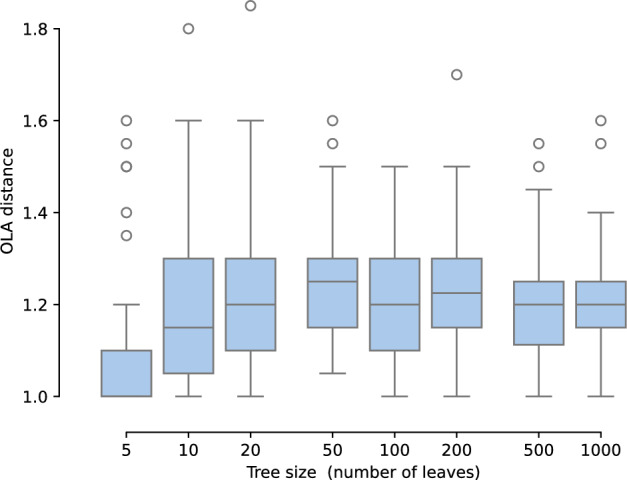
The expected OLA distance between a tree and its NNI neighbors. The size of the tree varies from 5 leaves to 1000 leaves. For each tree size, we generate 50 random focal trees, and for each focal tree we generate 20 random NNI neighbors. We then compute the average OLA distance between a focal tree and its sampled NNI neighbors. Theorem 4 states that the expected OLA distance is at most 4, while the simulated data suggests the actual expected distance is typically lower

**Fig. 5:**
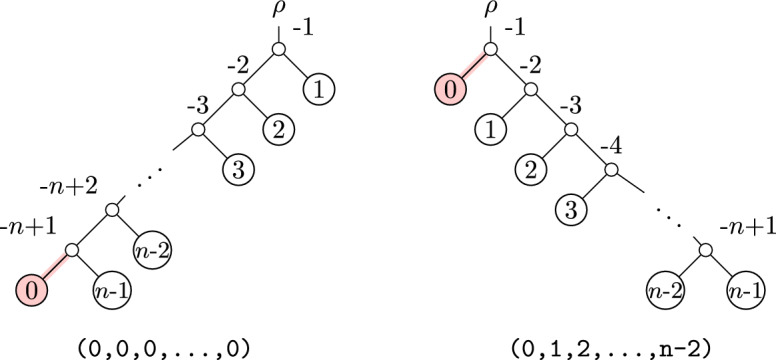
Two trees which differ by an SPR move, with large OLA distance

For a proof, see Appendix C. The bound in Theorem 4 is in agreement with computational experiment, as shown in Figures 4, 5.

#### Comparison to SPR distance

A common method for exploring the space of phylogenetic trees is through the use of subtree-prune-regraft (SPR) moves. A rooted SPR move consists of choosing a subtree to prune, and then choosing a location on the remaining tree at which to regraft the pruned subtree.

We introduce some standard terminology: the *height* of a rooted tree is the number of edges between the root node and the farthest leaf node. We use $\mathrm ht(T)$ to denote the height of the tree *T*.

##### Definition 5

Given a vertex *v* in a rooted tree *T*, the *height* of *v* is the distance from *v* to the root. The *height* of a (rooted) tree is the distance from the root to the farthest leaf.

##### Theorem 6

Suppose $T \in \widehat{\mathcal {RBT}}_n$ is a fixed phylogenetic tree, and let $T'$ be a tree which differs from *T* by a single SPR move. Then the OLA distance satisfies the upper bound

$$ d_{{{\,\textrm{OLA}\,}}}(T, T') \le 2 \cdot \mathrm ht(T), $$

where $\mathrm ht(T)$ denotes the height of *T*.

For a proof, see Appendix C. Note that as a special case, Theorem 6 also implies the bound $d_{{{\,\textrm{OLA}\,}}}(T, T') \le 2 \cdot \mathrm ht(T)$ for two trees that differ by a single NNI move.

**Fig. 6:**
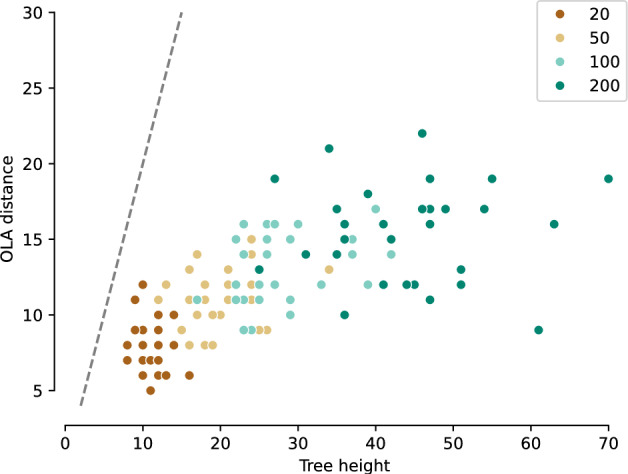
The maximum-sampled OLA distance between a tree and its SPR neighbors. The size of the tree varies from 20 leaves to 200 leaves, and is indicated by color. For each tree size, we generate 30 focal trees. For each focal tree, we sample 30 SPR neighbors, and plot the maximal OLA distance from the tree to one of its neighbors. Theorem 6 states that this OLA distance is bounded by 2 times the tree height, indicated by the dotted line. This bound is easily satisfied by the data in this simulation

Theorem 6 is supported by the following computational experiment, whose results are shown in Figure 6. We chose a random focal tree, sampled 30 of its SPR neighbors uniformly, and then computed the maximum OLA distance from the focal tree to these sampled neighbors. We then repeated this for a total of 30 focal trees, for each tree size in $\{20, 50, 100, 200\}$. This maximum OLA distance is plotted against the height of the initial focal tree. As suggested by the theorem, the resulting maximum OLA distance is smaller than the linear bound $2 \cdot \text {(tree height)}$.

### Further Comparison to SPR Distance

Rather than ask for the OLA distance between two trees that are SPR neighbors, we next ask for the SPR distance between two OLA neighbors. Given a tree *T*, we say $T'$ is obtained by a single *OLA move* from *T* if the OLA code $\Phi (T')$ differs from $\Phi (T)$ in exactly one entry. In other words, $\Phi (T')$ has a Hamming distance of one from $\Phi (T)$.

**Fig. 7:**
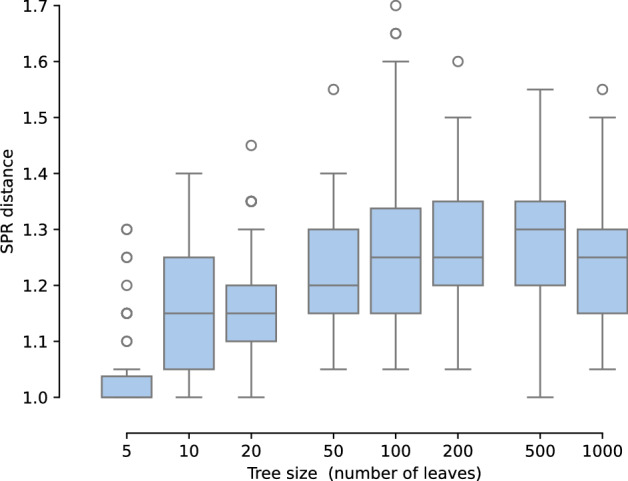
The average SPR distance between a tree and its OLA neighbors. For each tree size, 50 focal trees are randomly chosen. Then 20 OLA neighbors are sampled of each focal tree, and we average the SPR distances from these 20 trees to the focal tree to get one data point. The average SPR distances are all less than 2, in agreement with Theorem 7

#### Theorem 7

Suppose $T \in \widehat{\mathcal {RBT}}_n$ is a phylogenetic tree.

- If $T'$ is a tree that differs from *T* by a single OLA move, then the SPR distance satisfies the upper bound
  $$ d_{{{\,\textrm{SPR}\,}}}(T, T') \le \mathrm ht(T), $$
  where $\mathrm ht(T)$ denotes the height of *T*.
- If $T'$ is a random OLA neighbor of *T*, chosen uniformly, then the expected SPR distance satisfies the bound
  $$ \mathbb {E}(d_{{{\,\textrm{SPR}\,}}}(T, T')) < 2. $$

For a proof, see Appendix C. We ran the following experiment to show empirical agreement with Theorem 7 (b). For each tree size in the set $\{5, 10, 20, 50, 100, 200, 500, 1000\}$, we chose a random focal tree, sampled 20 random OLA neighbors, and then averaged the SPR distances from these neighbors to the focal tree. Then we repeated this for a total of 50 focal trees, for each tree size. The data is shown in Figure 7.

Markin and Anderson (2025) define a variation of the OLA distance, called the “corrected OLA distance”. They show that the minimum corrected OLA distance over all leaf orderings equals $|\textrm{MAAF}(T, T')| - 1$, where $\textrm{MAAF}$ denotes a maximum acyclic agreement forest (Bordewich and Semple 2005; Whidden et al. 2013). This connects to SPR distance via the following result.

#### Proposition 8

A pair of distinct trees $T, T'$ have corrected OLA distance equal to one under some leaf ordering if and only if $d_{{{\,\textrm{SPR}\,}}}(T, T') = 1$.

#### Proof

Bordewich and Semple (2005) showed that $d_{{{\,\textrm{SPR}\,}}}(T, T') = |\textrm{MAF}(T, T')| - 1$, where $\textrm{MAF}(T, T')$ denotes a maximum agreement forest. If the corrected OLA distance is one, then $|\textrm{MAAF}(T, T')| = 2$ (Markin and Anderson 2025). Since every MAAF is an agreement forest, we have $|\textrm{MAF}(T, T')| \le |\textrm{MAAF}(T, T')| = 2$. This gives $d_{{{\,\textrm{SPR}\,}}}(T, T') \le 1$. Since $T \ne T'$, we have $d_{{{\,\textrm{SPR}\,}}}(T, T') \ge 1$, and thus $d_{{{\,\textrm{SPR}\,}}}(T, T') = 1$. In the other direction, if $d_{{{\,\textrm{SPR}\,}}}(T, T') = 1$, then $|\textrm{MAF}(T, T')| = 2$. Every two-component MAF is acyclic because one of the components must contain the root, showing $|\textrm{MAAF}(T, T')| = 2$ and thus that the corrected OLA distance is 1 for some leaf ordering. $\square$

### Empirical Comparison to HOP Distance and SPR Distance

For an empirical comparison of OLA distance to HOP distance and SPR distance, see Appendix E. In this experiment, we measure how well the OLA distance, HOP distance, and RF distance correlate with the number of steps in a random SPR walk in tree space. We find that these correlations are all roughly comparable for trees on 100 leaves. However, if we replace the OLA and HOP distance with a “shuffled” version, by averaging the distances over 10 randomly chosen leaf orderings, then the correlation with SPR steps becomes noticeably better than the un-shuffled versions. Between the shuffled OLA distance and the shuffled HOP distance, this experiment also shows comparable correlation with SPR moves.

## Discussion

In this paper, we introduce a new encoding of phylogenetic trees: the “ordered leaf attachment” (OLA) code. This encoding is unique in being linear-time computable, and in having a simple description of the set of vectors corresponding to trees. The OLA encoding induces a distance on the set of trees, and we showed that this distance is in expectation approximately constant between NNI neighbors. We also showed that the OLA distance between a tree *T* and an SPR neighbor $T'$ is bounded above by the height of *T*, up to a constant factor.

There has been a long history of developing bijections between phylogenetic trees and other mathematical objects. In 1918, Prüfer (1918) defined an encoding, now known as the “Prüfer code”, of trees which have an ordering on all nodes (both leaf nodes and internal nodes), which proves combinatorially that the number of trees on *n* vertices is $n^{n - 2}$ (Cayley 1889). Erdös and Székely (1989) found a bijection between rooted phylogenetic trees and certain partitions of sets of integers. Diaconis and Holmes (1998, 2002) found a bijection from rooted phylogenetic trees on *n* leaves to perfect matchings on $2(n - 1)$ nodes.

There has been a recent surge of interest in developing embeddings of phylogenetic trees, initiated by the Phylo2Vec encoding introduced by Penn et al. (2024). This encoding has a similarly simple set of vectors corresponding to trees as the OLA encoding, however takes quadratic time to compute in general. For “Yule-type” trees, our OLA encoding agrees with the Phylo2Vec encoding, in both output and computational complexity.

Although both the Phylo2Vec and the OLA encoding have the same basic idea, OLA requires only linear time. The difference lies in the way that internal nodes are labeled. OLA uses negative integer labels which, because they are disjoint from the positive leaf labels, can be used persistently throughout the tree construction and labeling process. In contrast, for Phylo2Vec, internal nodes are labeled with positive integers, such that the internal node labels have to be regularly updated as the tree grows, to avoid conflicts with the leaf labels.

Using another approach, Chauve et al. (2025) introduce the LTS vector of a tree, for “labeling taxon sequence.” This approach takes linear time to compute. However, the construction is somewhat complex and the set of vectors corresponding to trees does not have a simple definition.

Both the Phylo2Vec and LTS papers also investigate distances between trees that are induced by the encoding. Specifically, Penn et al. (2024) examine how their Phylo2Vec-based Hamming distance relates to traditional tree metrics like the Subtree-Prune-Regraft (SPR) and Robinson–Foulds distances. Like for the OLA encoding, a small Phylo2Vec distance can correspond to a small or a large SPR distance, although experiments with SPR random walks show an approximately linear relationship between the two distances for small numbers of SPRs. Chauve et al. (2025) also introduce a “HOP distance” based on the LTS representation and analyze its relationship to NNI and SPR distances, showing that it often provides a better approximation of the SPR distance than the RF distance. A more detailed analysis between the OLA distance, Phylo2Vec distance, and HOP distance has been made in recent work (Linz et al. 2025).

In the original preprint version of our paper, we asked the question “Can we extend the OLA encoding to cover multifurcating tree topologies?” This question has been answered in the affirmative by Markin and Anderson (2025): they extend the OLA encoding to multifurcating trees and provide algorithms for preprocessing and optimally resolving such trees. In addition, they develop a modified version of the OLA distance that can be used to compute maximum acyclic agreement forests (MAAF) and hence find reticulation events. This work further establishes the OLA encoding as giving a topologically-relevant encoding of phylogenetic trees.

The OLA distance, Phylo2Vec distance, and HOP distance require an ordering of the set of taxa. Although this doesn’t pose a problem when calculating encodings on trees for a specific set of taxa, it does have disadvantages for the corresponding tree distances. Namely, classical tree distances only depend on a pair of leaf-labeled trees, while the Phylo2Vec, HOP, and OLA distances additionally depend on the ordering of the taxa. One can obtain an ordering-independent distance by taking the minimum or averaging over all possible orderings, but this may be expensive to compute. In the experiment described in Appendix E, we find that averaging the OLA and HOP distance over several random leaf orderings produces a distance that is better-behaved, in the sense of correlating better with SPR distance.

We note that while our definition of the OLA code is for rooted trees, it works equally well for unrooted trees. This is done by picking a leaf and making it the root. Although this requires an arbitrary choice, it introduces the same type of asymmetry as the requirement of choosing a leaf ordering for rooted trees, with the same associated drawbacks.

In the future, we will quantify the degree to which these encodings give natural descriptions of tree space. We also leave these more technical questions for future work:

(i) For a given tree shape (a rooted, bifurcating tree *without* leaf labels), for a randomly chosen leaf ordering, what is the expected number of negative entries in the corresponding OLA encoding? Is there any correlation with, e.g., tree balance?
(ii) Can we bound the variation of OLA distance caused by “shuffling”? Namely, for two trees with the same leaf taxa, how does the OLA distance change as we shuffle the leaf order, i.e. apply a permutation to the leaf labels on both trees together?

In the Appendix, we provide a suggested extension to trees with branch lengths, show a more involved example of an OLA encoding, provide the proofs deferred from Sect. 2.3 comparing OLA distance to standard tree distances, prove a theorem investigating random walks on trees using the OLA encoding, do an empirical analysis of OLA distance along random SPR and NNI walks, and end with an illustration of all the OLA encodings on three and four leaves.

## Methods

### OLA Encoding Details

Here we give a more thorough description, with proofs, of the OLA code of a rooted, binary, leaf-ordered tree.

#### Theoretical description

Given a tree with leaves labeled with $0, 1, 2, \ldots , n - 1$, we can imagine the tree being constructed from the empty tree by adding one leaf at a time in the specified order, as shown in Figures 1 and 2. As mentioned in the introduction, the OLA code of a rooted, bifurcating tree consists of the following main steps:

1. **Canonical internal label step**. When leaf node *i* is added, this creates a new internal node in the tree. Label this internal node as $-i$.
2. **OLA entry step**. When leaf node *i* is added, it is the sister to some previously-existing node (either a leaf node or an internal node). Record the sister leaf’s label as $a_i$, the *i*-th entry of the OLA encoding.

#### Practical description

Instead of following the steps above, where both the OLA encoding and canonical internal node labels are computed jointly at each step of the tree-construction process, we can make the following change. We find it more practical to compute all canonical internal labels in the first phase of the encoding, and then locate all sister-nodes to the leaf attachments in the second phase.

### Canonical Internal Node Labels

Here we describe a method of labeling the internal nodes of a phylogenetic tree, whose leaves have a chosen linear ordering. We assume this leaf ordering is encoded by assigning the labels $0, 1, 2, \ldots , n - 1$ to leaves.

#### Label algorithm

**Fig. 8:**
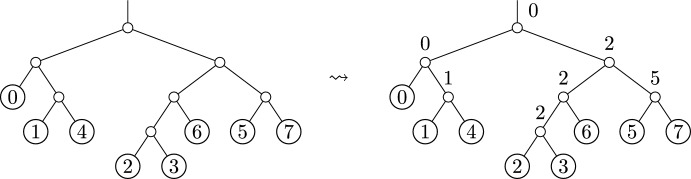
Assigning canonical internal node labels, step 1. Each internal node is labeled with its clade-founder label

**Fig. 9:**
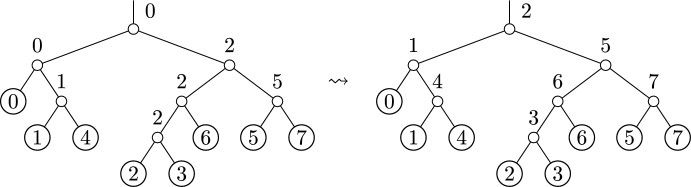
Assigning canonical internal node labels, step 2. On the right, each internal node is labeled with its clade-splitter label

To obtain canonical internal node labels, we apply the following steps:

1. Label internal nodes
  - Post-order (root-ward) traversal: label each node (either internal or leaf) by its “clade-founder” label. At leaves, this is equal to the leaf label. At internal nodes, this is equal to the minimum of the two children’s clade-founder labels. (See Figure 8.)
  - Pre-order (leaf-ward) traversal: label each internal node by its “clade-splitter” label. This is equal to the maximum of the two children’s clade-founder labels. (See Figure 9.)
  - Each internal node has canonical label $= -1\cdot$(clade-splitter label)

**Algorithm 1:**
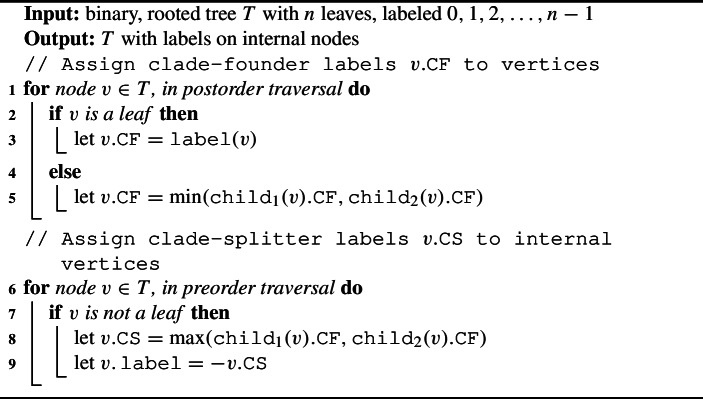
Canonical Internal Node Labels

##### Proposition 9

Given a binary, rooted tree with leaf labels $\{0, 1, 2, \ldots , n - 1\}$, the canonical internal node labeling (Algorithm 1) defines a bijective correspondence between the internal nodes and the labels $\{-1, -2, \ldots , -n + 1\}$.

##### Proof

We prove this by induction on *n*. If $n = 2$, then the unique internal node is assigned label $-1$.

Now suppose *T* is a tree with *n* leaves (with labels $0, \ldots , n-1$), where $n \ge 2$, and let $T^+$ denote a tree obtained by adding a leaf $v^+$ to *T*, with ${{\,\mathrm{\texttt {label}}\,}}(v^+) = n$. By the inductive hypothesis, we assume that the statement holds for *T*. The internal nodes of $T^+$ may be naturally identified with internal nodes of *T*, plus one additional node in $T^+$ which is the parent of leaf *n*. Suppose we call this parent node *p*. Since the statement holds for *T* by the inductive hypothesis, it suffices to show that

- the naturally identified internal nodes of *T* and $T^+$ have the same labels;
- *p* is assigned the label $-n$ in $T^+$.

For part (a), first observe that the clade-founder labels must agree on all nodes shared by *T* and $T^+$. Then to argue that the clade-splitter labels also agree, it suffices to consider the parent of the node *p* (in $T^+$). Call this parent node *w*. To compute ${w}.\texttt {CS}$ (in $T^+$), we consider ${p}.\texttt {CF}$ and the clade-founder of *w*’s other child. The other child is not affected by the change from *T* to $T^+$. For ${p}.\texttt {CF}$, its value is strictly smaller than *n*. Thus ${p}.\texttt {CF}$ is equal to the clade-founder label of its non-$v^+$-child, and the clade-founder label of this child agrees between *T* and $T^+$. This shows the label ${w}.\texttt {CS}$ in $T^+$ agrees with ${w}.\texttt {CS}$ in *T* as desired.

For part (b), according to Algorithm 1 line 8, we have ${p}.\texttt {CS} \ge n$ because *v* is a child of *p* and ${v}.\texttt {CF} = n$.

However, the reverse inequality ${p}.\texttt {CS} \le n$ also holds because the clade-founder labels satisfy ${*}.\texttt {CF} \le \max \{{{\,\mathrm{\texttt {label}}\,}}(w): w \}$. This shows that ${{\,\mathrm{\texttt {label}}\,}}(v) = -n$ as desired. $\square$

**Fig. 10:**
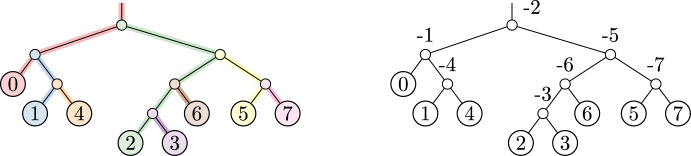
A tree with its branch decomposition highlighted by color (left) and its canonical internal node labels (right)

We can give another intuitive explanation for what we are recording with the canonical internal node labels. Given a leaf-ordered tree, which we imagine being constructed one leaf at a time, we can naturally “color” each of the edges according to the step in which it was added in the construction process (Figure 10). This coloring decomposes the tree into a collection of single-colored paths, each of which has one end at a leaf. If a path ends at the leaf *i*, then we label the other end (which must be an internal node) as $-i$. This perspective makes it clear that Proposition 9 holds, once we are convinced this agrees with the labels of Algorithm 1. Note: Figure 10 was inspired by a similar figure made in [ Chauve et al. (2025), Figure 2].

### Tree to OLA Code

To produce an OLA code from a labeled binary, rooted tree, we apply the following steps:

1. Apply canonical internal node labels (see Sect. 4.2).
2. Deconstruct the tree by removing leaves in reverse order; from leaf $n - 1$ to leaf 1.
  - Find leaf *i*, record the label of its sister node as $a_i$.
  - Remove leaf *i* and its parent, connecting the leaf’s sister node to its grandparent.

**Algorithm 2:**
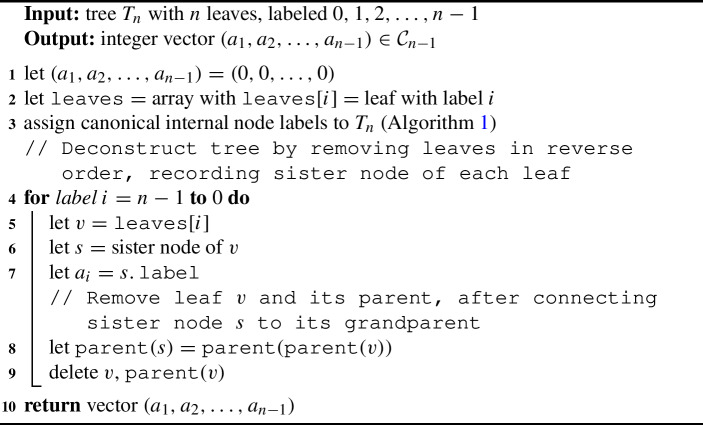
Tree to OLA Encoding

#### Theorem 10

The OLA encoding (Algorithm 2) defines a bijection

$$ \Phi : \widehat{\mathcal {RBT}}_n \rightarrow \mathcal {C}_{n - 1} $$

for every $$n \ge 2$$.

#### Proof

We prove this by induction on *n*. For $n = 2$, there is a single rooted, binary tree which is encoded as (0).

Now suppose that $n > 2$, and assume by induction that the OLA encoding is a bijection on trees with $n - 1$ leaves. Since $\widehat{\mathcal {RBT}}_n$ and $\mathcal {C}_{n - 1}$ both have cardinality equal to $(2n - 3)!!$, it suffices to show that the OLA encoding function is surjective. Choose an arbitrary integer vector $\textbf{v} = (a_1, a_2, \ldots , a_{n - 2}, a_{n - 1})$ in $\mathcal {C}_{n - 1}$. Let $\textbf{v}_{n - 1} = (a_1, \ldots , a_{n - 2})$ denote the vector obtained by deleting the last entry. By our inductive hypothesis, there is a tree $T_{n - 1}$ whose OLA encoding is $\textbf{v}_{n - 1}$. Using $T_{n - 1}$, we describe how to construct a tree *T* whose OLA encoding is $\textbf{v}$.

Suppose we assign canonical internal node labels (Algorithm 1) to $T_{n - 1}$. Then by Proposition 9, there is a unique internal node of $T_{n - 1}$ whose canonical label is $a_{n - 1}$. Let *T* be the tree obtained from $T_{n - 1}$ by subdividing the parent edge of the $a_{n - 1}$-node, and adding a new leaf under the new internal node. In *T*, the $a_{n - 1}$-node has the same canonical label, since the clade under this node doesn’t change. Thus the last entry of the OLA vector $\Phi (T)$ is equal to $a_{n - 1}$, as desired. Finally, all the previous entries in $\Phi (T)$ agree with $\textbf{v}$ by construction, due to Proposition 11. $\square$

In the following statement, if *T* is a leaf-ordered tree with *n* nodes, the notation $T|_k$ denotes the tree obtained by pruning all nodes from *T* that are larger than *k*, and “collapsing” nodes which have only one child in the result.

#### Proposition 11

The OLA encoding (Algorithm 2) is stable under last-leaf-deletion. In other words, if *T* has OLA vector ${(a_1,a_2,\ldots ,a_{n - 1})}$, then $T|_k$ has OLA vector ${(a_1,a_2,\ldots ,a_{k - 1})}$ for any $k < n$.

#### Proof

This is clear from the for-loop in lines 4-9 of Algorithm 2. $\square$

### OLA Code to Tree

To produce a tree from an OLA code, apply the following steps. Suppose we are given an OLA code $(a_1, a_2, \ldots , a_{n - 1})$.

1. Start with a rooted tree $T_1$ having a single leaf, labeled 0.
2. For each $i = 1, \ldots , n - 1$, construct $T_{i + 1}$ from $T_{i}$ as follows.
  - Suppose *i*-th entry is $a_i$. Find the node $n_i$ in tree $T_{i}$ labeled $a_i$.
  - Subdivide the parent-edge of $n_i$ with a new internal node. Give this new node the label $-i$.
  - Add a leaf node under $-i$, with label *i*. Call this resulting tree $T_{i + 1}$.
3. Return the final tree $T_{n}$ (forgetting internal labels).

**Algorithm 3:**
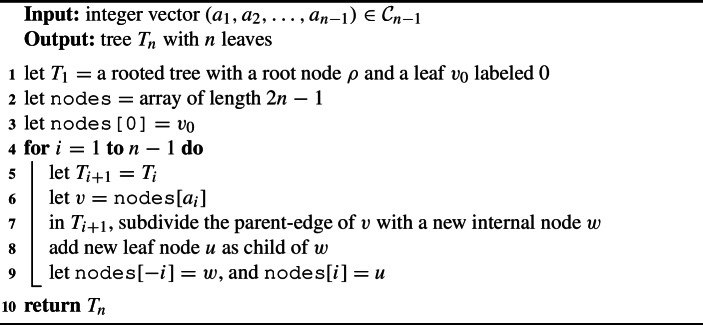
OLA to Tree Decoding Algorithm

#### Theorem 1

For any $n \ge 2$, the OLA encoding and decoding algorithms (Algorithms 2 and 3) define a pair of inverse bijections

2 $$\begin{aligned} \Phi : \widehat{\mathcal {RBT}}_n \rightarrow \mathcal {C}_{n - 1} \qquad \text {and}\qquad \Psi : \mathcal {C}_{n - 1} \rightarrow \widehat{\mathcal {RBT}}_n. \end{aligned}$$

#### Proof

We prove the claim by induction on *n*. For $n = 2$, both $\widehat{\mathcal {RBT}}_2$ and $\mathcal {C}_1$ contain a single element, and it is straightforward to check that we get bijections between them.

Now suppose $n \ge 3$. For an integer vector $\textbf{v} = (a_1, a_2, \ldots , a_{n - 1})$, we first claim that the last entry of $\Phi (\Psi (\textbf{v}))$ is equal to $a_{n - 1}$. In the tree $\Psi (\textbf{v})$, by construction (Algorithm 3 lines 6-9) a neighborhood of leaf $n - 1$ looks like the figure below. 
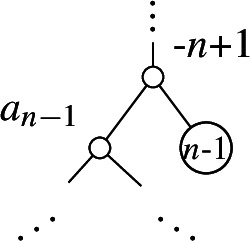
 Then by construction of the OLA encoding, the last entry of $\Phi (\Psi (\textbf{v}))$ only depends on this local picture, and results in last entry $a_{n - 1}$, as claimed.

To confirm that the remaining entries $a_1, \ldots , a_{n - 2}$ of $\textbf{v}$ agree with $\Phi (\Psi (\textbf{v}))$, we apply our inductive hypothesis, along with the property that $\Phi$ is stable under last-leaf-deletion, Proposition 11. $\square$

### Running Time

We show that converting a tree to an OLA code, and vice versa, takes linear time in the number of leaves.

#### Theorem 13

The process of assigning canonical internal node labels to a tree with *n* leaves has time complexity *O*(*n*).

#### Proof

Algorithm 1 consists of two traversals of the tree, and each step of the traversal requires a constant-time computation. $\square$

#### Theorem 2

The OLA encoding and decoding algorithms have linear time complexity, in terms of the number of leaves.

#### Proof

We separate the encoding and decoding parts of the argument.

- First suppose we encode a tree on *n* leaves. The first step of the OLA encoding (Algorithm 2) is assigning canonical internal node labels, which takes linear time (Theorem 13).
  The next step iterates through the leaves of the tree, in reverse order from their given ordering (i.e. largest-to-smallest). This can be achieved efficiently by constructing a label-to-leaf array in a single tree traversal (not included in the pseudocode in Algorithm 2), which takes linear time. Finding the sister node (line 6) takes constant time, as does the tree modification operation (lines 8-9).
- The OLA decoding (Algorithm 3) consists of initializing a linear-length array and a single linear-length loop, so it suffices to explain why each iteration of the loop is constant-time. This is clear because the tree modification operations (lines 7-8) take constant time, as does updating the nodes array (line 9). Copying the previous tree in the loop (line 5) can be done by reference. The condition that $-i< a_i < i$ for vectors in $\mathcal {C}_{n - 1}$ guarantees that in line 6, *v* is a well-defined node of tree $T_{i + 1}$, rather than an uninitialized array entry.

$$\square $$

### Code Details

We have implemented the linear-time OLA encoding and decoding algorithms in Python, using the ETE3 library (Huerta-Cepas et al. 2016) and minimal other dependencies. The package is available at https://github.com/matsengrp/ola-encoding.

## Figures and Tables

**Fig. 11:**
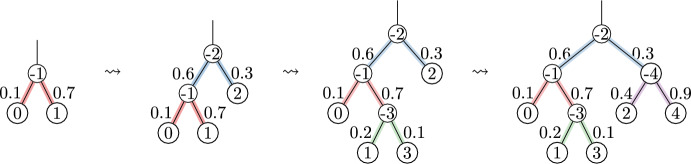
OLA encoding of a binary tree with branch lengths

**Fig. 12:**
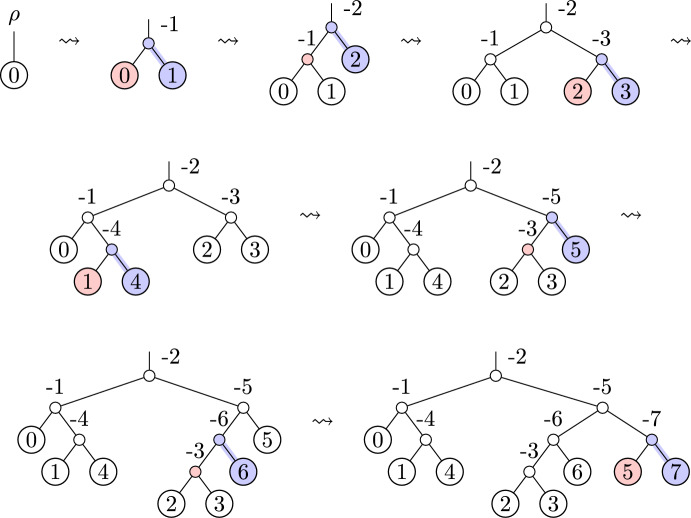
Constructing the tree with OLA code $\texttt {(0,-1,2,1,-3,-3,5)}$

**Fig. 13:**
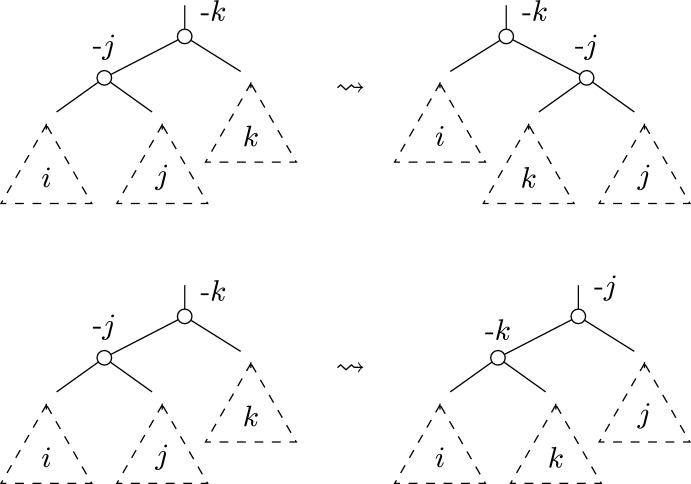
Trees differing by an NNI move. For the purposes of the proof of Theorem 4, the left tree is *T* and the right tree is $T'$

**Fig. 14:**
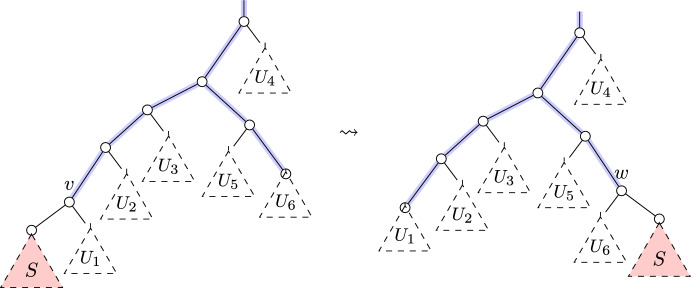
A rooted SPR move, which prunes and regrafts the subtree *S* highlighted in red. The central tripod is highlighted in blue

**Fig. 15:**
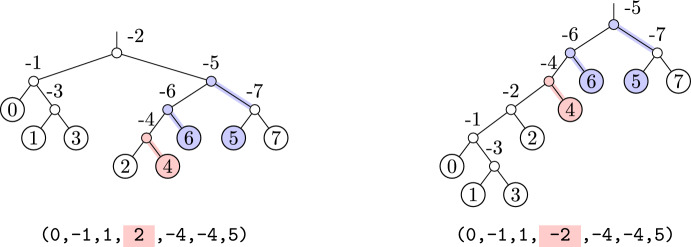
Two trees $T, T'$ whose OLA codes differ by exactly one entry, $a_4$. The SPR distance between these trees satisfies $d_{{{\,\textrm{SPR}\,}}}(T, T') \le 3$, by pruning and regrafting the subtrees under the three highlighted edges. The edge above the ($-4$)-th subtree is highlighted red, while the edges above the ($-j$)-th subtrees for each index with entry $a_j = -4$ are highlighted blue

**Fig. 16:**
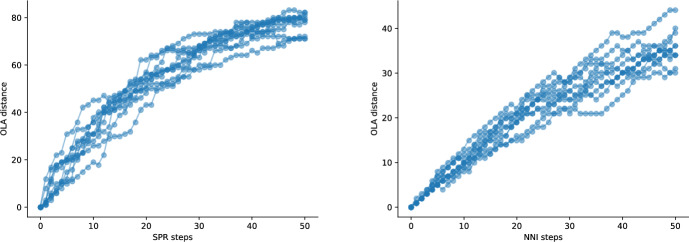
Comparing OLA distance to starting tree for random SPR walks on trees with 100 leaves (left), and random NNI walks on trees (right). Here each walk consists of choosing a random starting tree and applying a sequence of 50 random SPR moves (NNI moves). The plot shows the OLA distance between the starting tree and each tree along the walk, for 10 SPR walks (NNI walks)

**Fig. 17:**
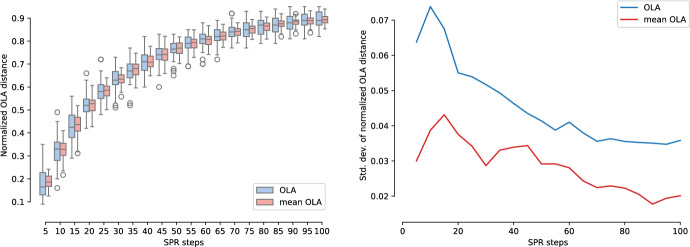
We compare the OLA distance with the mean OLA distance (left), where the mean OLA is averaged over 10 randomly chosen leaf orderings. Each box plot represents 50 data points, corresponding to 50 starting trees on 100 leaves. For each starting tree, we choose a sequence of 100 random SPR moves, and compute the distance from each starting tree to the tree obtained after $k = 5, 10, \ldots , 100$ SPR moves are applied. We also compute the standard deviation of the OLA and mean OLA distances (right), over the 50 sampled tree pairs that differ by *k* SPR steps. Both plots show that there is less variation in the mean OLA distance, in comparison with OLA distance

**Fig. 18:**
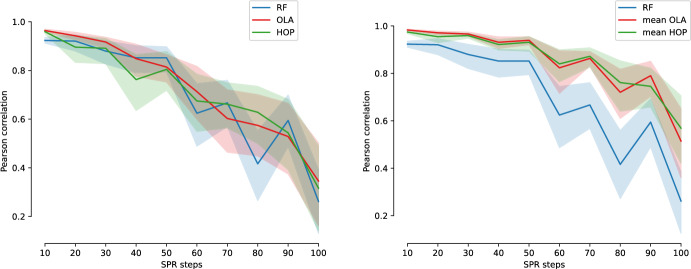
Pearson correlation between number of SPR moves and efficiently-computable distance on trees. Here we choose 50 focal phylogenetic trees on 100 leaves, and for each focal tree apply 100 random SPR moves. The correlation is computed on bins of size 10 each

**Fig. 19:**
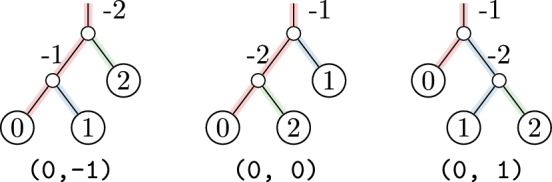
All phylogenetic trees on three leaves, with their OLA encodings

**Fig. 20:**
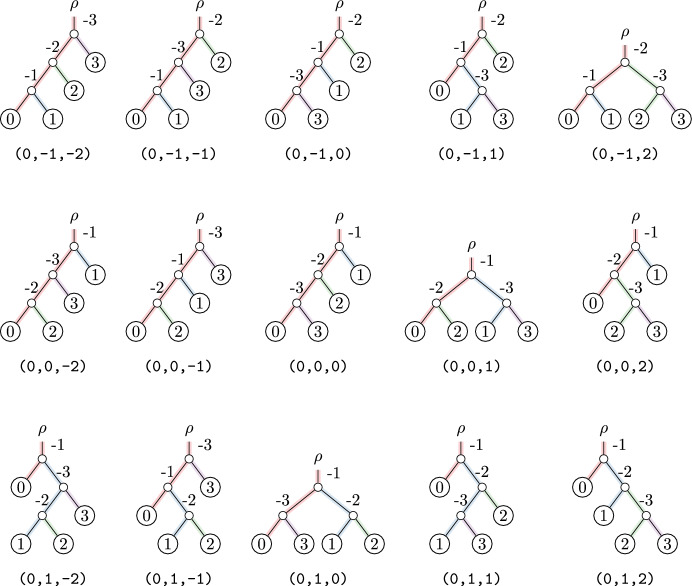
All phylogenetic trees on four leaves, with their OLA encodings

## OLA Code for Trees with Branch Lengths

The OLA encoding can be easily extended to encode phylogenetic trees with branch lengths. This can be achieved by adding two additional rows underneath the original OLA code, with entries consisting of positive real numbers. These real numbers prescribe the lengths of the two new branches that form when a new leaf is attached.

Rather than spell out all the details here, we illustrate a possible branch-length OLA encoding with an example. Figure 11 shows a tree with branch lengths, as it is constructed one leaf at a time. This tree is encoded by the array  where the colors highlight which lengths correspond to which branches.

Alternatively, the tree with branch lengths could be encoded in “one-line format” as

## Example OLA Encoding

We demonstrate the construction with a more involved example of a phylogenetic tree, compared to the example in the introduction, shown in Figure 12.

## Comparison of OLA Distance with NNI and SPR Distances: Proofs

In this section, we include proofs of results stated in the main text that compare the OLA distance with SPR distance and NNI distance.

### Theorem 4

Suppose *T* is a fixed phylogenetic tree in $\widehat{\mathcal {RBT}}_n$, and let $T'$ be a random tree which differs from *T* by an NNI move, chosen uniformly. Then the expectation of the OLA distance between *T* and $T'$ has the constant upper bound

$$ \mathbb {E}(d_{{{\,\textrm{OLA}\,}}}(T, T')) \le 4. $$

### Proof

Consider trees *T* and $T'$ which differ by an NNI move.

Suppose the NNI move is achieved by first contracting an edge in *T* which lies between nodes with canonical labels $-j$ and $-k$, with $-k$ on top (Figure 13). After the NNI move, in $T'$, the modified edge either still has label $-k$ on top, or switches to have label $-j$ on top. In the first case, where the top-label stays the same, we claim that $d_{{{\,\textrm{OLA}\,}}}(T, T')$ is exactly 1. This is because the only change in the OLA code is at entry $a_j$.

In the second case, where the top-label changes in $T'$, let $c_k$ denote the number of entries in the OLA encoding of *T* which are $-k$’s. We claim that $d_{{{\,\textrm{OLA}\,}}}(T, T')$ is bounded above by $1 + c_k$. Indeed, if some entry of the OLA encoding changes between *T* and $T'$, then that entry is either $a_j$ or $a_k$ (which accounts for the “1”), or else some entry $a_\ell$, for a leaf which attaches just above the NNI-modified edge. In the latter case, we have $a_\ell = -k$ in the OLA encoding of *T* and $a_\ell = -j$ in the encoding of $T'$.

Thus, accounting for both cases,

$$ \mathbb {E}(d_{{{\,\textrm{OLA}\,}}}(T, T')) \le \mathbb {E}(1 + c_k) $$

where $c_k = c_{k(T')}$ depends on the NNI move chosen (which determines $T'$). In a tree with *n* leaves, there are $2n - 4$ possible NNI moves. For each index *k* there are at most four NNI moves with “upper node” $-k$. In other words, for each fixed *k* there are at most four NNI-neighbors $T'$ such that $k(T') = k$. Thus,

$$\begin{aligned} \mathbb {E}(1 + c_{k(T')})&= 1 + \frac{1}{2n - 4} \sum _{T'} c_{k(T')} \\&\le 1 + \frac{4}{2n - 4} \sum _{k = 1}^n c_k \\&= 1 + \frac{4n - 4}{2n - 4} \end{aligned}$$

where the first sum varies over $T'$ which are one NNI move away from *T*. In the last line, we use the fact that $\sum _{k = 1}^n c_k \le n - 1$, since the OLA encoding of *T* has $n - 1$ entries. Finally, we have that

$$ 1 + \frac{4n - 4}{2n - 4} \le 4 \qquad \text {when } n \ge 4, $$

so the claim is proved. $\square$

### Theorem 6

Suppose $T \in \widehat{\mathcal {RBT}}_n$ is a fixed phylogenetic tree, and let $T'$ be a tree which differs from *T* by a single SPR move. Then the OLA distance satisfies the upper bound

$$ d_{{{\,\textrm{OLA}\,}}}(T, T') \le 2 \cdot \mathrm ht(T), $$

where $\mathrm ht(T)$ denotes the height of *T*.

### Proof

Suppose the SPR move from *T* to $T'$ prunes a subtree *S* below vertex *v*, and regrafts *S* below vertex *w*. Consider the “tripod” of *T* consisting of the paths between *v*, *w*, and the root $\rho$ of *T*. (To be precise, *w* is not a node of *T*; but we are referring to the child node of the edge which gets subdivided by *w* after the SPR move.) We may decompose *T* into a union of the vertices and edges on this tripod, and the collection of subtrees that branch off of the tripod. Let us denote by $U_1, U_2, \ldots , U_k$ the subtrees branching off of the tripod, in addition to the moved subtree *S*. See Figure 14 for an illustration, where the central tripod is highlighted in blue.

When comparing the OLA codes $\Phi (T)$ and $\Phi (T')$, the canonical labeling remains the same within the interior of *S* and of each subtree $U_i$, and can only change for vertices on the tripod. Thus we can have at most one OLA entry change within each of these subtrees. As a result, we have the bound

$$ d_{{{\,\textrm{OLA}\,}}}(T, T') \le k + 1. $$

Finally, we note that $k + 1$ is bounded above by $2 \cdot \mathrm ht(T)$. $\square$

### Proof of Theorem 7

- First we introduce some terminology. For a tree with *n* leaves and ordered leaf labels, the ($-i$)-*th subtree* refers to the following. Suppose we apply canonical leaf labels to internal nodes (see Sect. 4.2). Then for each *i* in the range $1 \le i \le n - 1$, there is a unique internal node with the label $-i$. This internal node has two subtrees below it, and exactly one of those subtrees contains leaf *i*. We call this subtree the ($-i$)-th subtree.
  Now returning to the theorem, suppose we change the *i*-th entry of the OLA code $\Phi (T)$ to obtain $\Phi (T')$. Let *k* denote the number of $-i$’s that appear in the OLA code $\Phi (T)$. Then we can get from *T* to $T'$ in $1 + k$ SPR moves, as follows: first, prune and regraft the ($-i$)-th subtree to its new location in $T'$, then for each *j* with $a_j = -i$, prune and regraft the ($-j$)-th subtree to its new location. This argument shows that
  $$\begin{aligned} d_{{{\,\textrm{SPR}\,}}}(T, T') \le 1 + k . \end{aligned}$$
  See Figure 15 for an illustration, where the 4-th entry is changed, and the number of $-4$’s that appear in the OLA code is $k = 2$.
  Finally, we use the fact that the height of the tree satisfies $1 + k \le \mathrm ht(T)$. This is because $1 + k$ is a lower bound for the distance from leaf *i* to the root, while by definition $\mathrm ht(T)$ is an upper bound for this distance.
  Note that when the OLA codes of *T* and $T'$ differ at the *i*-th entry, this argument gives the more precise bound
  3 $$\begin{aligned} d_{{{\,\textrm{SPR}\,}}}(T, T') \le 1 + (\text {number of (-i)'s that appear in }\Phi (T)) . \end{aligned}$$
- Each tree on *n* leaves has $(n - 1)(n - 2)$ OLA neighbors, since the *i*-th entry may be changed to one of $2i - 2$ different options, for $i = 1, 2, \ldots , n - 1$. Let $n_i$ be the number of entries in $\Phi (T)$ that are equal to $-i$. We have
  $$\begin{aligned}&\mathbb {E}(d_{{{\,\textrm{SPR}\,}}}(T, T')) \\&\quad = \sum _{i = 1}^{n - 1} \mathbb {P}(\Phi (T') \text { changes }\,\, a_i-\text {entry})\; \mathbb {E}(d_{{{\,\textrm{SPR}\,}}}(T, T') \;|\; a_i -\text {entry changed}) \\&\quad \le \sum _{i = 1}^{n - 1} \mathbb {P}(a_i\text {-entry changed}) (1 + n_i) \qquad \qquad \text {by applying } (3)\\&\quad = 1 + \sum _{i = 1}^{n - 1} \mathbb {P}(\Phi (T') \text { changes}\,\, a_i-\text {entry}) \cdot n_i \\&\quad = 1 + \sum _{i = 1}^{n - 1} \frac{2i - 2}{(n - 1)(n - 2)} \cdot n_i . \end{aligned}$$
  Now we can write $n_i$ in terms of indicator functions as $\displaystyle n_i = \sum _{j = i + 1}^{n - 1} 1\!\!1(a_j = -i)$. By using this in the above expression, and then exchanging the order of summations, we obtain
  $$\begin{aligned} \mathbb {E}(d_{{{\,\textrm{SPR}\,}}}(T, T'))&= 1 + \sum _{i = 1}^{n - 1} \frac{2i - 2}{(n - 1)(n - 2)} \left( \sum _{j = i + 1}^{n - 1} 1\!\!1(a_j = -i) \right) \\&= 1 + \frac{1}{(n - 1)(n - 2)} \sum _{j = 2}^{n - 1} \sum _{i = 1}^{j - 1} 1\!\!1(a_j = -i) \cdot (2i - 2) . \end{aligned}$$
  The inner term $\displaystyle \sum _{i = 1}^{j - 1} 1\!\!1(a_j = -i) (2i - 2)$ is strictly bounded above by $(2j - 2)$, since clearly $a_j$ can only take on one value. Therefore
  $$\begin{aligned} \mathbb {E}(d_{{{\,\textrm{SPR}\,}}}(T, T')) < 1 + \frac{1}{(n - 1)(n - 2)} \sum _{j = 1}^{n - 1} (2j - 2) = 2 . \end{aligned}$$
  as claimed. $$\square $$

## Tree Distance and Random Walks

### Generating Random Trees

A common problem in phylogenetics is to efficiently traverse the space of all (binary) phylogenetic trees on a given number of leaves.

The OLA code allows us to define a random walk on the set of rooted, binary trees $\mathcal {RBT}_n$ by applying the OLA decoding function to a Gibbs random walk on the set of vectors $\mathcal {C}_{n - 1}$. By “Gibbs random walk,” we mean one step in the random walk consists of choosing a random index *i*, and changing the entry at *i* to any valid integer uniformly, so that the outcome lies in $\mathcal {C}_{n - 1}$. It is natural to ask: how quickly do we expect this random walk to become well-dispersed over tree space?

#### Theorem 17

Let $t^*$ denote the number of steps in a random OLA walk after which the resulting probability distribution on $\mathcal {RBT}_n$ is uniform. Then $\mathbb {E}(t^*) = \Theta (n \log n)$.

#### Proof

This is a direct application of the expected time of the coupon collection problem [ Motwani and Raghavan (1995), Chapter 3.6].

The OLA code consists of $n - 1$ entries, and each step in an OLA walk (Gibbs walk) consists of choosing one entry and changing the value of that entry to a uniformly-random chosen value (out of the set of valid values, $-i< a_i < i$). The state of the walk is independent of the starting point exactly when every entry between $a_2$ and $a_{n - 1}$ has been chosen to be “mixed.” (We ignore $a_1$, since this entry is always 0.) This situation is equivalent to the coupon collector problem: suppose there are $n - 2$ coupon types, and one wishes to collect all types. Each coupon has a uniformly-random type. Then a standard result of probability theory states that the expected number of coupons needed is $\Theta ((n - 2) \log (n - 2))$, which is asymptotically equivalent to $n \log n$. $\square$

Diaconis and Holmes (2002) consider a different method for randomly perturbing binary trees that also mixes in $n \log n$ time, up to a constant factor. Earlier, Aldous (2000) considers randomly perturbing binary trees by pruning and regrafting a single leaf at each step. He shows that this process mixes more slowly, in polynomial time between $O(n^2)$ and $O(n^3)$.

### OLA Distance Along Random SPR Walks and NNI Walks

In Sect. 2.3, we investigated bounds on the OLA distance of two trees which differ by a single NNI move or a single SPR move. In general, we may also want to consider phylogenetic trees that are farther apart in NNI- or SPR-distance.

In Figure 16, we consider running an SPR-random-walk (respectively, NNI-random-walk) in which a tree is repeatedly perturbed by a uniformly selected random SPR or NNI move. We can observe in the figures that the OLA distance increases approximately linearly with slope one for NNI walks, while the OLA distance for SPR walks initially increases with slope greater than one, then gradually levels off to slope one by around 30 steps. In both cases, the OLA distance levels off after sufficiently many moves. This leveling-off likely corresponds to a leveling-off of the SPR distance as well, in which a collection of random SPR moves can be compressed into a smaller number of SPR moves. For example, once the random walk reaches the SPR diameter of tree space, the SPR distance cannot increase any further.

Compare to [ Penn et al. (2024), Figure 5] for the Phylo2Vec distance.

## Leaf Shuffling and OLA Distance

In this section, we investigate how OLA distance is affected by changing the ordering on the leaf taxa. We then compare the OLA distance with HOP distance and RF distance. The comparison of OLA and HOP distance is also studied in Linz et al. (2025).

In Figure 17, we run the following experiment to investigate the effect of leaf shuffling. We select 50 random trees on 100 leaves as starting trees for applying a sequence of SPR moves. For each starting tree, we choose a sequence of 100 random SPR moves, and calculate the OLA distance $d_{{{\,\textrm{OLA}\,}}}(T, T')$ between each starting tree *T* and the tree $T'$ resulting from applying *k* SPR moves, for $k = 5, 10, \ldots , 100$. For each number *k* of SPR moves, we compute the standard deviation of the 50 sampled OLA distances.

We then repeat the procedure for 10 randomly chosen orderings of the leaf taxa and average the resulting distances $d_{{{\,\textrm{OLA}\,}}}(T, T')$ for the same tree pairs. (The distance $d_{{{\,\textrm{OLA}\,}}}$ implicitly depends on a choice of leaf ordering.) We call the resulting average the “mean OLA distance” or “shuffled OLA distance” between *T* and $T'$.

This data is shown in Figure 17. In the figure, all the OLA distances are normalized by dividing by the number of leaves (100). The figures show that averaging over several leaf orderings (mean OLA) results in a decrease in the variance, compared to usual OLA, over pairs of trees at a fixed number of SPR moves apart. The variance also tends to decrease as the number of SPR moves increases. Figure 17 can be compared to [ Chauve et al. (2025), Figures S1 and S2] for the HOP distance.

**Table 1:** Pearson correlation between number of SPR moves and an efficiently-computable tree distance. The computed tree distance is either Robinson–Foulds (RF), OLA with a fixed leaf ordering, OLA averaged over 10 randomly-chosen leaf orderings (mean OLA), HOP with a fixed leaf ordering, or HOP averaged over 10 randomly-chosen leaf orderings (mean HOP). The column “10” refers to the bin of $\{1, 2, \ldots , 10\}$ SPR moves, etc. These correlations are averages over the 50 random SPR walks

SPR moves	10	20	30	40	50	60	70	80	90	100
RF	0.923	0.921	0.880	0.852	0.852	0.624	0.667	0.416	0.595	0.212
OLA	0.964	0.942	0.917	0.848	0.814	0.713	0.603	0.574	0.529	0.346
mean OLA	**0.983**	**0.971**	**0.966**	**0.931**	**0.939**	0.823	0.863	0.720	**0.790**	0.514
HOP	0.959	0.896	0.891	0.762	0.804	0.675	0.661	0.628	0.544	0.316
mean HOP	0.974	0.955	0.959	0.922	0.931	**0.840**	**0.872**	**0.761**	0.745	**0.569**

### OLA Distance and HOP Distance

Since OLA distance depends on an ordering of the leaf taxa, while SPR distance is ordering-independent, we might expect that averaging the OLA distance over several choices of ordering will better correlate with SPR distance (as the leaf-order-dependent irregularities are “evened-out”).

We test this hypothesis in the following experiment, as suggested by Chauve et al. (2025) where the HOP distance is compared with the RF distance. We sample several random SPR walks on trees with 100 leaves, and use the number of SPR steps and a proxy for the SPR distance from the starting tree. For each random walk, we compute the following distances from the starting tree to the tree obtained from some number of random SPR moves:

4 $$\begin{aligned} \begin{gathered} \text {RF distance, OLA distance, HOP distance,}\\ \text {mean OLA distance, mean HOP distance.} \end{gathered} \end{aligned}$$

The Robinson–Foulds (RF) distance (Robinson and Foulds 1981) is used as a baseline, and this distance does not depend on a leaf ordering. The HOP distance is defined in Chauve et al. (2025), and is dependent on a leaf ordering. The “mean OLA” and “mean HOP” distances refer to taking the mean over 10 randomly-chosen leaf orderings.

In more detail, in this experiment we choose 50 random focal trees on 100 leaves. For each focal tree, we apply a sequence of 100 random SPR moves to obtain a random SPR walk. For each tree in the SPR walk, we compute the distance $d_*(T, T')$ from the tree to the starting focal tree, where $d_*$ is one of the five distances (4). These distances are separated into bins of length 10, i.e. for moves $\{1, \ldots , 10\}$, moves $\{11, \ldots , 20\}$, etc. Finally, for each bin, we compute the Pearson correlation between the measured distance and the number of SPR moves. A higher correlation indicates better agreement between the given distance and the SPR distance. (The number of applied SPR moves serves as a proxy for SPR distance, which is computationally expensive to find.)

In Figure 18, we see that the measured correlation of OLA distance and HOP distance to SPR steps is roughly comparable to RF distance, when using only a single leaf ordering (left side). However, when we average over 10 randomly-chosen leaf orderings (Figure 18, right side), we see that the correlation of the mean OLA distance and mean HOP distance with SPR steps is significantly improved over RF distance. Both mean OLA and mean HOP distances perform comparably for this metric. The correlation data is also shown in Table 1.

## OLA Code for Trees on Three or Four Leaves

We demonstrate the OLA encoding by showing the OLA code for all rooted, bifurcating trees on three and four leaves, in Figures 19 and 20.
